# Evaluation and comparison of six GRACE models for the stratification of undifferentiated chest pain in the emergency department

**DOI:** 10.1186/s12872-020-01476-3

**Published:** 2020-04-25

**Authors:** Wen Zheng, Guangmei Wang, Jingjing Ma, Shuo Wu, He Zhang, Jiaqi Zheng, Feng Xu, Jiali Wang, Yuguo Chen

**Affiliations:** 1 Department of Emergency Medicine and Chest Pain Center, Qilu Hospital of Shandong University, No.107, Wen Hua Xi Road, Jinan, 250012 Shandong China; 2 Clinical Research Center for Emergency and Critical Care Medicine of Shandong Province, Institute of Emergency and Critical Care Medicine of Shandong University, Qilu Hospital of Shandong University, Jinan, China; 3 Key Laboratory of Emergency and Critical Care Medicine of Shandong Province, Key Laboratory of Cardiopulmonary-Cerebral Resuscitation Research of Shandong Province, Qilu Hospital of Shandong University, Jinan, China; 4 The Key Laboratory of Cardiovascular Remodeling and Function Research, Chinese Ministry of Education, The State and Shandong Province Joint Key Laboratory of Translational Cardiovascular Medicine, Chinese Ministry of Health and Chinese Academy of Medical Sciences, Qilu Hospital of Shandong University, Jinan, China

**Keywords:** Chest pain, GRACE, HEART, TIMI, Stratification

## Abstract

**Background:**

The Global Registry of Acute Coronary Events (GRACE) score is recommended for stratifying chest pain. However, there are six formulas used to calculate the GRACE score for different outcomes of acute coronary syndrome (ACS), including death (Dth) or composite of death and myocardial infarction (MI), while in hospital (IH), within 6 months after discharge (OH6m) or from admission to 6 months later (IH6m). We aimed to perform the first comprehensive evaluation and comparison of six GRACE models to predict 30-day major adverse cardiac events (MACEs) in patients with acute chest pain in the emergency department (ED).

**Methods:**

Patients with acute chest pain were consecutively recruited from August 24, 2015 to September 30, 2017 from the EDs of two public hospitals in China. The 30-day MACEs included death, acute myocardial infarction (AMI), emergency revascularization, cardiac arrest and cardiogenic shock. The correlation, calibration, discrimination, reclassification and diagnostic accuracy at certain cutoff values of six GRACE models were evaluated. Comparisons with the History, ECG, Age, Risk Factors, and Troponin (HEART) and Thrombolysis in Myocardial Infarction (TIMI) scores were conducted.

**Results:**

A total of 2886 patients were analyzed, with 590 (20.4%) patients experiencing outcomes. The GRACE (IHDthMI), GRACE (IH6mDthMI), GRACE (IHDth), GRACE (IH6mDth), GRACE (OH6mDth) and GRACE (OH6mDthMI) showed positive linear correlations with the actual MACE rates (r ≥ 0.568, *P* < 0.001). All these models had good calibration (Hosmer-Lemeshow test, *P* ≥ 0.073) except GRACE (IHDthMI) (*P* < 0.001). The corresponding C-statistics were 0.83(0.81,0.84), 0.82(0.81,0.83), 0.75(0.73,0.76), 0.73(0.72,0.75), 0.72(0.70,0.73) and 0.70(0.68,0.71), respectively, first two of which were comparable to HEART (0.82, 0.80–0.83) and superior to TIMI (0.71, 0.69–0.73). With a sensitivity ≥95%, GRACE (IHDthMI) ≤81 and GRACE (IH6mDthMI) ≤79 identified 868(30%) and 821(28%) patients as low risk, respectively, which were significantly better than other GRACEs and HEART ≤3(22%). With a specificity ≥95%, GRACE (IHDthMI) > 186 and GRACE (IH6mDthMI) > 161 could recognize 12% and 11% patients as high risk, which were greater than other GRACEs, HEART ≥8(9%) and TIMI ≥5(8%).

**Conclusions:**

In this Chinese setting, certain strengths of GRACE models beyond HEART and TIMI scores were still noteworthy for stratifying chest pain patients. The validation and reasonable application of appropriate GRACE models in the evaluation of undifferentiated chest pain should be recommended.

## Background

Chest pain and related symptoms are the most common reasons for patients to present to the emergency department (ED) [1, 2], and present extremely heterogeneous with a wide spectrum of underlying conditions ranging from lethal diseases such as acute myocardial infarction (AMI) to minor acute problems such as intercostal neuralgia. Ruling in or ruling out high-risk conditions in a timely manner is of great importance and a great challenge [3–6]. Furthermore, the majority of undifferentiated acute chest pain patients are low risk and do not require further invasive tests or admission [6, 7]. Therefore, risk stratification for chest pain patients at EDs has been recommended in several guidelines [6, 8] to not only identify as many true low-risk patients as possible but also avoid missing major adverse cardiac events (MACEs).

The Global Registry of Acute Coronary Events (GRACE) score is an objective prediction tool for definite acute coronary syndrome (ACS), incorporating age, vital signs, kidney function, ECG and troponin levels [9]. This tool has been validated for risk stratification of individuals with acute chest pain [10–17]. In particular, the 0 h/3 h algorithm with the GRACE score incorporated into is recommended (Class I, Level B) for risk stratification and rule-out of AMI in patients with suspected non-ST-elevation ACS by 2015 European Society of Cardiology (ESC) guideline [6].

However, there are six formulas used to calculate the GRACE score for different outcomes, including those for predicting in-hospital death [9], in-hospital death or myocardial infarction (MI) [18], death within 6 months after discharge [19], death or MI within 6 months after discharge, death from admission to 6 months later [20], and death or MI from admission to 6 months later [20]. None of these formulas are specific for rule-out/rule-in of high-risk conditions in patients with undifferentiated chest pain presenting to the ED. The GRACE models have been compared with other scores for stratifying undifferentiated chest pain, such as the History, ECG, Age, Risk Factors, and Troponin (HEART) score and the Thrombolysis in Myocardial Infarction (TIMI) score [21, 22]. Generally, GRACE was inferior to the HEART score, and the most common GRACE score applied was the one for predicting in-hospital death [11–16]. The questions of why this model is selected and whether it is the most appropriate one remain unanswered. No study has comprehensively assessed these scores in detail in chest pain patients. Therefore, the superiority of certain GRACE scores remains unclear.

Using a range of model performance indices, we aimed to evaluate the performance of six GRACE models and compare their discrimination, reclassification and diagnostic accuracy with those of HEART and TIMI scores to rule out/rule in 30-day MACEs among acute chest pain patients presenting to the ED.

## Methods

### Study design

This is a secondary analysis of a previous cohort. We prospectively collected data through an observational study of acute chest pain patients from August 24, 2015 to September 30, 2017 in EDs of two public hospitals in China, the urban ED of the Qilu Hospital of Shandong University (a university-affiliated teaching hospital) and the rural ED of the People’s Hospital of Wenshang County. This study was approved by the ethics committees of the collaborating hospitals. Written informed consent was obtained from all participants.

### Patient enrolment

Patients were consecutively recruited if they were aged 18 or older and had acute nontraumatic chest pain and troponin tests. Acute symptoms of myocardial ischaemia or an ischaemic equivalent, such as epigastric discomfort, dyspnoea or fatigue, were also considered as chest pain according to the American Heart Association case definitions [23].

To assess the performance of risk scores for the stratification of non-ST-elevation chest pain, patients with ST-elevation myocardial infarction (STEMI) were excluded. Other exclusion criteria for analysis included patients unable or unwilling to provide informed consent.

### Data collection and measurements

Clinical information was extracted from the medical records and collected through patient interviews by research assistants using a standardized case report form (CRF); the variables were in accordance with the international standards [23]. Demographics, risk factors, previous medical history, symptom characteristics, physical examination, vital signs, troponin values, laboratory tests, triage, treatments and outcomes were covered.

Patients participated in follow-up telephone interviews at 30 days after enrolment, and information about MACEs and hospital attendances was collected.

### Risk score calculations

Methods and formulas used to calculate the GRACE risk scores have been described in detail previously [24], including all 6 models for predicting in-hospital death (IHDth), in-hospital death or MI (IHDthMI), death within 6 months after discharge (OH6mDth), death or MI within 6 months after discharge (OH6mDthMI), death from admission to 6 months later (IH6mDth), and death or MI from admission to 6 months later (IH6mDthMI) (Table 1). These scores were calculated retrospectively using the prospectively obtained data. An ECG with ST depression (new horizontal or down-sloping ST depression ≥0.05 mV in two contiguous leads) or ST elevation (new ST elevation at the J point in two contiguous leads with the cut-points: ≥0.1 mV in all leads other than leads V2-V3 where the following cut points apply: ≥0.2 mV in men ≥40 years; ≥0.25 mV in men < 40 years, or ≥ 0.15 mV in women) [25] was defined as ischaemic ST deviation. Two independent cardiologists interpreted the ECGs blinded to the clinical data, troponin levels and events. Discrepancies were evaluated by a third cardiologist. The troponin results from the first blood sample arranged by emergency physicians in their daily work were used to calculate scores. The 99th percentile of the upper reference limit (URL) was used as the cutoff for determining positivity. In-hospital PCI and in-hospital CABG were assigned a score of 0 because the GRACE models were used to stratify chest pain patients immediately after arrival at ED and the PCI or CABG performed during admission were not applicable. In emergency care practice, not all GRACE predictor variables can be collected completely, especially the serum creatinine test (not routinely assessed) and the Killip class (not rated in patients without AMI). Here, we performed two kinds of assessment: one was based on the complete GRACE variables with the creatinine value and Killip class assigned as zero if absent; the other was based on the deletion of creatinine and Killip class from all observations using mini-GRACE, which has been introduced in the development of NICE guideline 94 and validated through a large MINAP registry of patients with ACS [26, 27]. The HEART and TIMI scores were calculated according to previous studies (Supplementary Table 1) [22, 28].

**Table 1:** Variables included in six GRACE models

Scores	Abbreviation	Variables
**GRACE**		
In-hospital death	GRACE (IHDth)	Age, SBP, Pulse, Cr level, Killip class, CA at admission, Positive cTn, ST deviation
In-hospital death or MI	GRACE (IHDthMI)	Age, SBP, Pulse, Cr level, Killip class, CA at admission, Positive cTn, ST deviation
Death within 6 months after discharge	GRACE (OH6mDth)	Age, SBP, Pulse, Cr level, Positive cTn, ST depression, Past MI, Past CHF, In-hospital PCI
Death or MI within 6 months after discharge	GRACE (OH6mDthMI)	Age, Positive cTn, Past MI, Past CHF, In-hospital CABG
Death from admission to 6 months later	GRACE (IH6mDth)	Age, SBP, Pulse, Cr level, Killip class, CA at admission, Positive cTn, ST deviation
Death or MI from admission to 6 months later	GRACE (IH6mDthMI)	Age, SBP, Cr level, Killip class, CA at admission, Positive cTn, ST deviation

*CA* cardiac arrest, *CHF* congestive heart failure, *Cr* creatinine, *cTn* cardiac troponin, *CABG* coronary artery bypass grafting, *GRACE* Global Registry of Acute Coronary Events, *MI* myocardial infarction, *PCI* percutaneous coronary intervention, *SBP* systolic blood pressure

### Clinical outcomes

The primary outcome was the composite endpoint of MACEs within 30 days, including death from all causes, AMI (index and subsequent), emergency revascularization, cardiac arrest and cardiogenic shock. The diagnosis of AMI was performed according to the third universal definition of MI, as a detection of the rise and/or fall in cardiac biomarkers with at least one value above the 99th percentile of URL and with symptoms or ECG changes or imaging indicative of new ischaemia [25]. Cardiogenic shock was defined as persistent (> 30 min) systolic blood pressure (SBP) of less than 90 mmHg and/or cardiac index < 2.2 L/min/m^2^ secondary to cardiac dysfunction, requiring intravenous inotropic or mechanical support [23]. Two senor cardiologists from the clinical events committee adjudicated the MACEs independently using all available clinical records, and discrepancies were evaluated by a third senior physician. If patients were lost to follow-up, a local death registry was used to supplement the survival status.

### Statistical analysis

Continuous variables are presented as the mean (standard deviation), and categorical variables are presented as the number of cases (percentage). Baseline characteristics between the MACE and no MACE groups were compared using t tests for continuous variables and chi-square (χ^2^) tests for categorical variables. Pearson product-moment correlation was used as “r” to describe the direction and quantify the strength of the linear association between GRACE scores and the incidence of MACEs in individuals with chest pain. The calibration was evaluated using the Hosmer-Lemeshow goodness-of-fit test (HLT). Low HLT χ^2^ and *P* value > 0.05 illustrate agreement between observed and predicted probabilities of an event and good model fit. Discrimination of scores was assessed by the area under the curve (AUC) of receiver operating characteristic (ROC) curves. An AUC of 0.8 ~ 0.9 is considered excellent and 0.7 ~ 0.8 acceptable [29]. Taking into account the implicit correlation between the curves of these scores, we used the Delong test to compare any two AUCs [30]. Reclassification was performed to assess how well a risk score improved predictions compared with another one based on category-free net reclassification improvement (NRI) and absolute integrated discrimination improvement (IDI). Diagnostic accuracy with 95% confidence intervals (CIs) of the different scores was determined, including sensitivity, specificity, negative predictive value (NPV) and positive predictive value (PPV). To guarantee the safety of discharging low-risk patients, cutoff values of each model to obtain sensitivities of at least 95%, 98% and 99% were identified. High-risk cutoff values were set at a specificity of ≥95% or ≥ 90%. The sensitivity and specificity of different strategies were compared using the McNemar test, while proportions of patients classified as low-risk and high risk, NPV and PPV were compared using the χ2 test. A *P* value of less than 0.05 (two-sided significance testing) was considered statistically significant in the analysis. All statistical analyses were performed using SAS V.9.4 (SAS Institute Inc., Cary, North Carolina, USA) or MedCalc V.18.11.3 (MedCalc Software, Ostend, Belgium).

## Results

### Study population

**Fig. 1:**
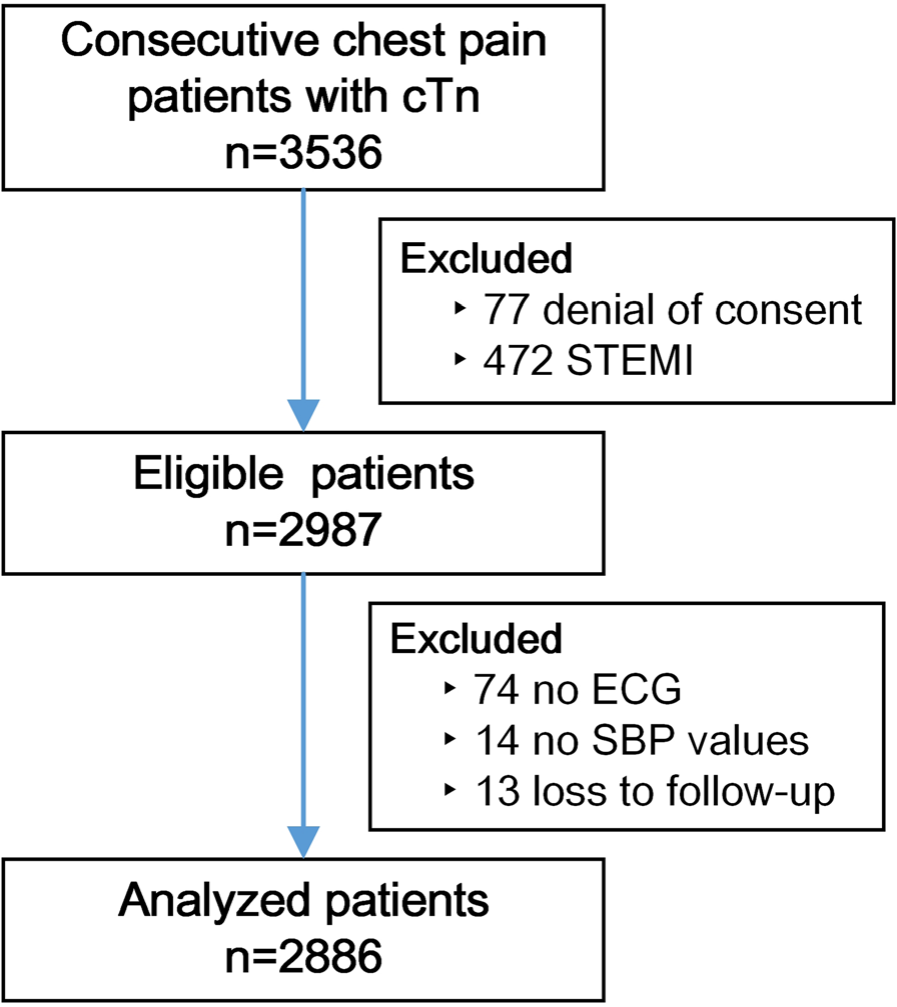
Study flowchart. *cTn* cardiac troponin, *ECG* electrocardiography, *STEMI* ST-segment elevation myocardial infarction, *SBP* systolic blood pressure

A total of 3536 patients with acute nontraumatic chest pain and initial cTn tests presented in the participating EDs from August 24, 2015 to September 30, 2017. Some patients were excluded due to denial of informed consent (77) and diagnosis of STEMI (472). There were 88 patients with insufficient information to calculate the GRACE scores, including 74 due to no initial ECG and 14 due to no SBP values. For 13 surviving patients, follow-up contacts were unsuccessful. Eventually, 2886 patients remained for analysis (Fig. 1). Baseline characteristics and initial evaluation between patients with and without 30-day MACEs are compared in Table 2. Patients with 30-day MACEs tended to be older, be male, have a higher burden of risk factors and have significantly higher GRACE scores than those without 30-day MACEs (*P* < 0.001).

**Table 2:** Baseline characteristics of the study population

	Total (*n* = 2886)	MACE (*n* = 590)	No MACE (*n* = 2296)	*P* value
**Demographics**				
Age (y), mean (SD)	64 (13.5)	67 (12.0)	63 (13.7)	< 0.001
BMI (kg/m^2^), mean (SD)	25 (3.6)	25 (3.3)	25 (3.7)	0.557
Male, n (%)	1447 (50.1)	354 (60.0)	1093 (47.6)	< 0.001
**Risk factors, n (%)**				
Current smoker	483 (16.7)	148 (25.1)	335 (14.6)	< 0.001
Diabetes	676 (23.4)	180 (30.5)	496 (21.6)	< 0.001
Hypertension	1725 (59.8)	400 (67.8)	1325 (57.7)	< 0.001
Hyperlipidaemia	281 (9.7)	62 (10.5)	219 (9.5)	0.478
Family history of premature CAD	455 (15.8)	79 (13.4)	376 (16.4)	0.076
**Medical history, n (%)**				
MI	602 (20.9)	159 (26.9)	443 (19.3)	< 0.001
CAG with stenosis ≥50%	694 (24.0)	141 (23.9)	553 (24.1)	0.924
PCI	511 (17.7)	86 (14.6)	425 (18.5)	0.026
CABG	73 (2.5)	29 (4.9)	44 (1.9)	< 0.001
Heart failure	76 (2.6)	27 (4.6)	49 (2.1)	0.001
CRF	48 (1.7)	20 (3.4)	28 (1.2)	< 0.001
PAD	5 (0.2)	4 (0.7)	1 (0)	0.007
Stroke	396 (13.7)	89 (15.1)	307 (13.4)	0.281
**Presentation vital signs, mean (SD)**				
SBP (mmHg)	149 (27.7)	147 (30.2)	150 (27.0)	0.021
DBP (mmHg)	84 (16.2)	85 (18.5)	84 (15.6)	0.038
Pulse (bpm)	81 (19.1)	83 (23.1)	80 (17.9)	0.007
**Initial cTn positive, n (%)**	489 (16.9)	370 (62.7)	119 (5.2)	< 0.001
**Initial ST deviation, n (%)**	1301 (45.1)	415 (70.3)	886 (38.6)	< 0.001
**Scores, mean (SD)**				
GRACE (IHDth)	110 (34.0)	135 (34.4)	104 (30.8)	< 0.001
GRACE (IHDthMI)	121 (53.9)	174 (52.6)	107 (45.0)	< 0.001
GRACE (OH6mDth)	99 (30.4)	118 (29.4)	94 (28.7)	< 0.001
GRACE (OH6mDthMI)	101 (34.2)	120 (34.6)	96 (32.4)	< 0.001
GRACE (IH6mDth)	88 (30.7)	109 (30.5)	83 (28.4)	< 0.001
GRACE (IH6mDthMI)	107 (43.5)	148 (42.7)	96 (36.8)	< 0.001

*BMI* body mass index, *bpm* beats per minute, *CABG* coronary artery bypass grafting, *CAD* coronary artery disease, *CAG* coronary angiography, *CRF* chronic renal failure, *cTn* cardiac troponin, *DBP* diastolic blood pressure, *GRACE* Global Registry of Acute Coronary Events, *MACE* major adverse cardiac events, *MI* myocardial infarction, *PAD* peripheral arterial disease, *PCI* percutaneous coronary intervention, *SBP* systolic blood pressure, *SD* standard deviation

### Outcomes

There were 590 (20.4%) chest pain patients with adjudicated MACEs in 30 days after presentation, including 52 patients (1.8%) who died from all causes, 549 (19.0%) with index AMI, 24 (0.8%) with subsequent AMI, 10 (0.3%) who underwent emergency revascularization, 32 (1.1%) who experienced cardiac arrest and 32 (1.1%) who experienced cardiogenic shock.

### Correlation between GRACE scores and actual event rates

**Fig. 2:**
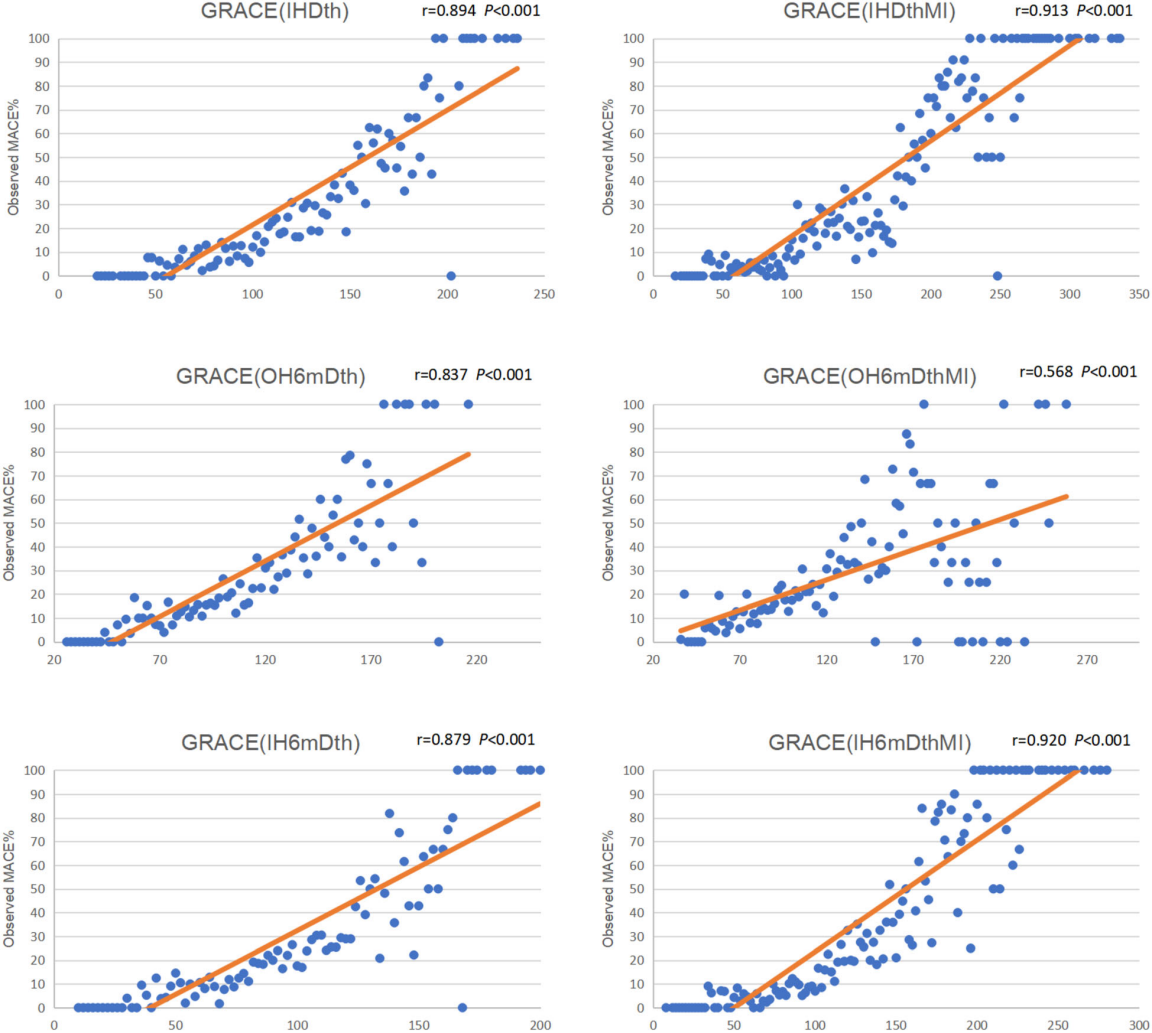
Pearson product-moment correlation between GRACE scores and actual event rates. *GRACE* Global Registry of Acute Coronary Events, *MACE* major adverse cardiac events

All six GRACE models showed good positive linear correlation with the actual MACE rates in patients with undifferentiated chest pain (Fig. 2). The GRACE (IHDthMI) and GRACE (IH6mDthMI) exhibited very strong relationships, with r values of 0.913 and 0.920, respectively (*P* < 0.001).

### Agreement between observed and predicted probabilities of an event

**Fig. 3:**
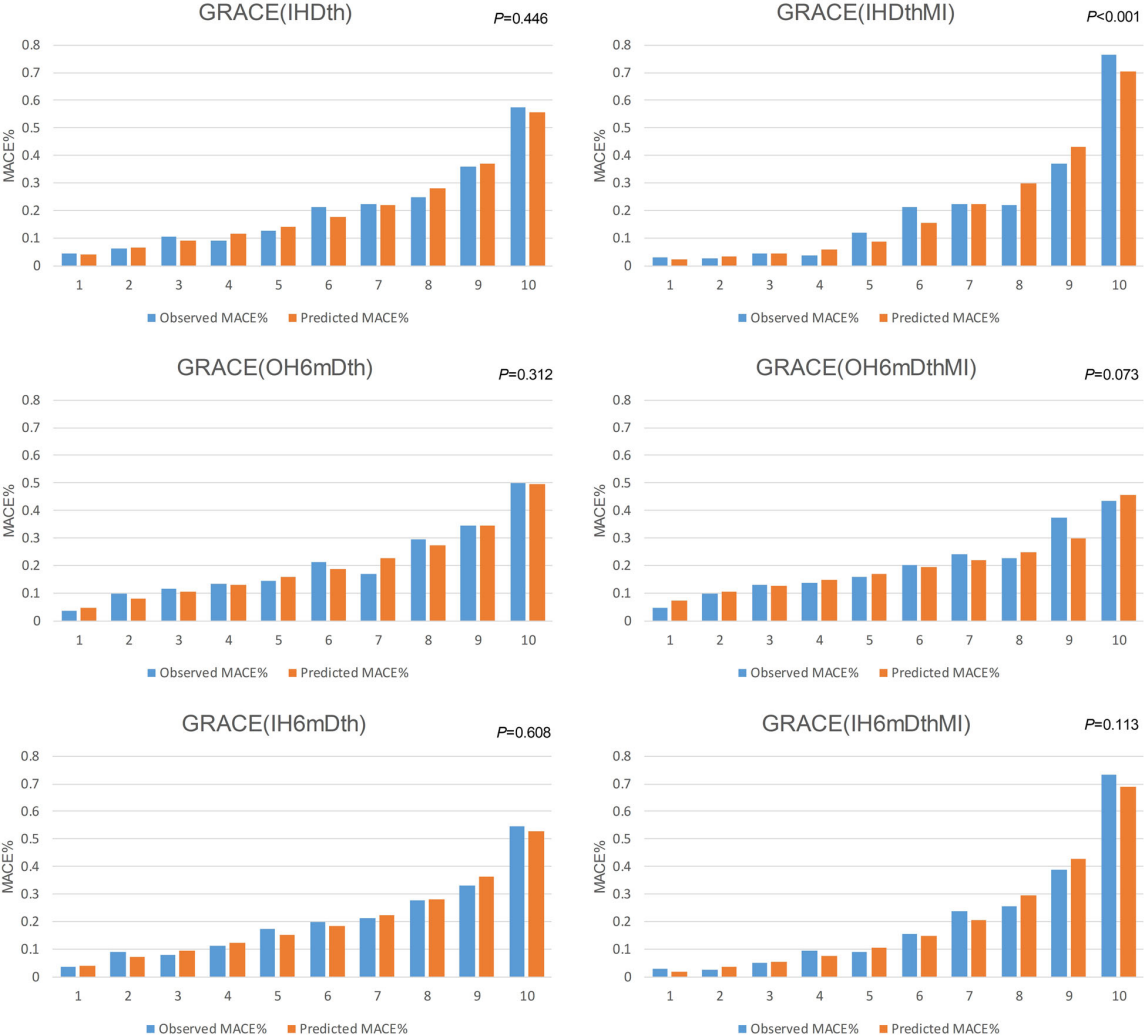
Hosmer-Lemeshow goodness-of-fit tests of six GRACE models. *GRACE* Global Registry of Acute Coronary Events, *MACE* major adverse cardiac events

As shown in Fig. 3, the predicted probabilities of an event were much close to the observed event rates across deciles of five GRACE models. And the HLT *P* values for the GRACE (IH6mDthMI), GRACE (IHDth), GRACE (IH6mDth), GRACE (OH6mDth) and GRACE (OH6mDthMI) were 0.113, 0.446, 0.608, 0.312 and 0.073, respectively. However, the *P* value of GRACE (IHDthMI) was < 0.001.

### Discrimination

**Fig. 4:**
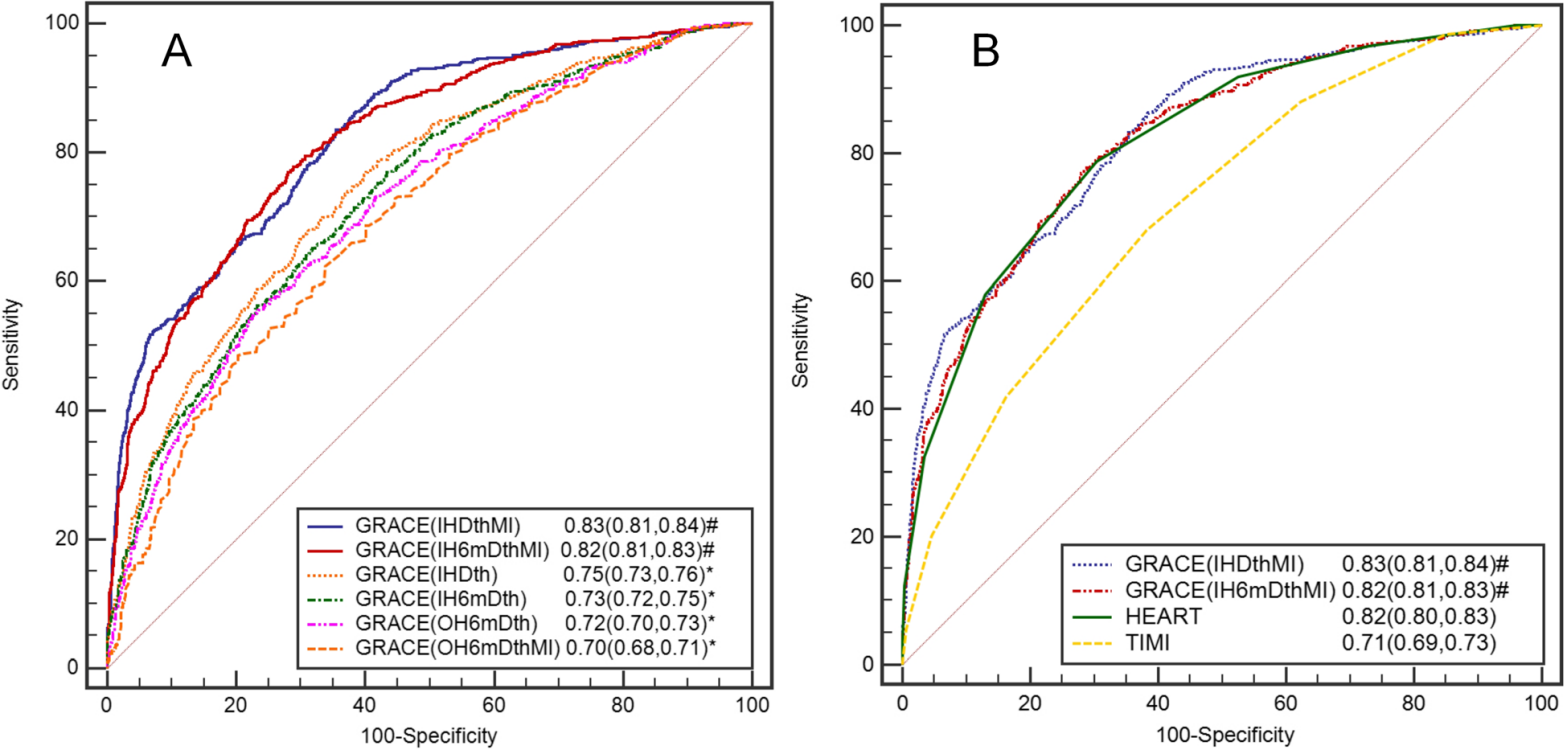
Receiver operating characteristic curves of six GRACE models (**a**), HEART and TIMI scores (**b**) for the prediction of MACE within 30 days. *GRACE* Global Registry of Acute Coronary Events, *HEART* History, ECG, Age, Risk factors, Troponin, *MACE* major adverse cardiac events, *TIMI* Thrombolysis in Myocardial Infarction. * Significantly different from the HEART score. # Significantly different from the TIMI score

The ROC curves of all the GRACE models, HEART and TIMI scores are depicted in Fig. 4. The AUCs of GRACE (IHDthMI), GRACE (IH6mDthMI), GRACE (IHDth), GRACE (IH6mDth), GRACE (OH6mDth) and GRACE (OH6mDthMI) were 0.83 (0.81, 0.84), 0.82 (0.81, 0.83), 0.75 (0.73, 0.76), 0.73 (0.72, 0.75), 0.72 (0.70, 0.73) and 0.70 (0.68, 0.71), respectively. The AUCs of GRACE (IHDthMI) and GRACE (IH6mDthMI) were equal to the C-statistic of HEART score at 0.82 (0.80, 0.83) and superior to the other GRACE models and the TIMI score (0.71, 0.69–0.73) (*P* < 0.001). The C-statistics of the GRACE models and the HEART and TIMI scores in each participating hospital are presented in Supplementary Table 2.

### Reclassification

As shown in Table 3, the GRACE (IHDthMI) improved risk classifications of chest pain patients with positive NRI and IDI over the other GRACE models and the TIMI score (*P* < 0.001), while it presented comparable ability to the HEART score. The same trend was seen in the GRACE (IH6mDthMI) model.

**Table 3:** The reclassification measurements of the GRACE (IHDthMI) and GRACE (IH6mDthMI) compared with other scores

	NRI (95%CI)	*P* value	IDI (95%CI)	*P* value
GRACE (IHDthMI) vs				
GRACE (IH6mDthMI)	0.296 (0.206, 0.385)	< 0.001	0.021 (0.014, 0.027)	< 0.001
GRACE (IHDth)	0.970 (0.893, 1.046)	< 0.001	0.132 (0.121, 0.143)	< 0.001
GRACE (IH6mDth)	0.899 (0.821, 0.977)	< 0.001	0.150 (0.137, 0.162)	< 0.001
GRACE (OH6mDth)	0.827 (0.747, 0.908)	< 0.001	0.173 (0.157, 0.188)	< 0.001
GRACE (OH6mDthMI)	0.791 (0.709, 0.873)	< 0.001	0.204 (0.185, 0.223)	< 0.001
HEART	0.044 (−0.046, 0.135)	0.338	0.026 (0.006, 0.046)	0.012
TIMI	0.868 (0.786, 0.950)	< 0.001	0.177 (0.159, 0.196)	< 0.001
GRACE (IH6mDthMI) vs				
GRACE (IHDth)	1.158 (1.081, 1.235)	< 0.001	0.111 (0.102, 0.121)	< 0.001
GRACE (IH6mDth)	1.079 (1.003, 1.154)	< 0.001	0.129 (0.119, 0.139)	< 0.001
GRACE (OH6mDth)	0.970 (0.891, 1.049)	< 0.001	0.152 (0.139, 0.165)	< 0.001
GRACE (OH6mDthMI)	0.908 (0.828, 0.989)	< 0.001	0.183 (0.166, 0.200)	< 0.001
HEART	−0.115 (−0.205, −0.025)	0.013	0.005 (− 0.015, 0.024)	0.636
TIMI	0.773 (0.689, 0.857)	< 0.001	0.156 (0.138, 0.175)	< 0.001

*CI* confidence interval, *GRACE* Global Registry of Acute Coronary Events, *HEART* History, ECG, Age, Risk factors, Troponin, *IDI* integrated discrimination improvement, *NRI* net reclassification improvement, *TIMI* Thrombolysis in Myocardial Infarction

### Rule-out and rule-in of events

For discharging low-risk patients safely with sensitivity ≥95% (as shown in Table 4), GRACE (IHDthMI) ≤81 and GRACE (IH6mDthMI) ≤79 could identify 868 (30%) and 821 (28%) patients as low risk, respectively, which were significantly better than other GRACEs and a HEART score ≤ 3 (22%). Additionally, GRACE (IHDthMI) ≤81 and GRACE (IH6mDthMI) ≤79 ruled out MACEs with an NPV of 0.967 (0.955, 0.979) and 0.965 (0.952, 0.977), respectively, exceeding other GRACEs and comparable to a HEART score ≤ 3 with an NPV of 0.970 (0.957, 0.983). If the sensitivity was set at ≥98%, the proportion of patients identified as low risk would decrease to 14% for GRACE (IHDthMI) ≤64 and GRACE (IH6mDthMI) ≤61 with no change in the superiority to HEART ≤2 (11%) and TIMI =0 (12%) (Supplementary Table 3). If the sensitivity was improved up to ≥99%, the proportions of low-risk patients would drop below 10% for GRACEs, which were still significantly greater than a HEART score ≤ 1 (3%) (Supplementary Table 3).

**Table 4:** Performance of different models in terms of diagnostic accuracy at cutoff values with certain sensitivity and specificity levels

Scores and cutoff values	No. (%)	Sensitivity (95%CI)	NPV (95%CI)	Specificity (95%CI)	PPV (95%CI)
**Sensitivity ≥ 95%**	**Low-risk**				
GRACE (IHDthMI) ≤81	868 (30%)*	0.951 (0.933,0.968)	0.967 (0.955,0.979)	0.365 (0.346,0.385)*	0.278 (0.258,0.298)
GRACE (IH6mDthMI) ≤79	821 (28%)*	0.951 (0.933,0.968)	0.965 (0.952,0.977)	0.345 (0.326,0.364)*	0.272 (0.252,0.291)
GRACE (IHDth) ≤79	520 (18%)*	0.951 (0.933,0.968)	0.944 (0.925,0.964)*	0.214 (0.197,0.231)*	0.237 (0.220,0.254)
GRACE (IH6mDth) ≤57	466 (16%)*	0.954 (0.937,0.971)	0.942 (0.921,0.963)*	0.191 (0.175,0.207)*	0.233 (0.216,0.249)
GRACE (OH6mDth) ≤66	408 (14%)*	0.956 (0.939,0.972)	0.936 (0.913,0.960)*	0.166 (0.151,0.182)*	0.228 (0.211,0.244)*
GRACE (OH6mDthMI) ≤68	438 (15%)*	0.956 (0.939,0.972)	0.941 (0.919,0.963)*	0.179 (0.164,0.195)*	0.230 (0.214,0.247)
HEART ≤3	639 (22%)	0.968 (0.954,0.982)	0.970 (0.957,0.983)	0.270 (0.252,0.288)	0.254 (0.236,0.272)
**Specificity ≥ 95%**	**High-risk**				
GRACE (IHDthMI) > 186	357 (12%)*#	0.437 (0.397,0.477)* #	0.869 (0.856,0.882)* #	0.957 (0.949,0.965)	0.723 (0.676,0.769) #
GRACE (IH6mDthMI) > 161	328 (11%)*#	0.386 (0.347,0.426)* #	0.858 (0.845,0.872) #	0.956 (0.948,0.965)	0.695 (0.645,0.745) #
GRACE (IHDth) > 159	234 (8%)	0.234 (0.200,0.268)*	0.830 (0.815,0.844)	0.958 (0.950,0.966)	0.590 (0.527,0.653)*
GRACE (IH6mDth) > 133	218 (8%)*	0.203 (0.171,0.236)*	0.824 (0.809,0.838)*	0.957 (0.949,0.966)	0.550 (0.484,0.616)*
GRACE (OH6mDth) > 144	219 (8%)*	0.198 (0.166,0.230)*	0.823 (0.808,0.837)*	0.956 (0.947,0.964)	0.534 (0.468,0.600)*
GRACE (OH6mDthMI) > 151	189 (7%)*	0.154 (0.125,0.183)* #	0.815 (0.800,0.830)*	0.957 (0.949,0.966)	0.481 (0.410,0.553)*
HEART ≥8	272 (9%)#	0.325 (0.288,0.363) #	0.848 (0.834,0.862) #	0.965 (0.958,0.973) #	0.706 (0.652,0.760) #
TIMI ≥5	224 (8%)*	0.202 (0.169,0.234)*	0.823 (0.809,0.838)*	0.954 (0.946,0.963)*	0.531 (0.466,0.597)*

*CI* confidence interval, *GRACE* Global Registry of Acute Coronary Events, *HEART* History, ECG, Age, Risk factors, Troponin, *NPV* negative predictive value, *PPV* positive predictive value, *TIMI* Thrombolysis in Myocardial Infarction

*Significantly different from the HEART score

# Significantly different from the TIMI score

Regarding ruling in MACEs with specificity ≥95% (Table 4), GRACE (IHDthMI) > 186 and GRACE (IH6mDthMI) > 161 could recognize 12% and 11% of patients as high risk, respectively, which were more than a HEART ≥8 (9%) and a TIMI ≥5 (8%). The sensitivities of GRACE (IHDthMI) > 186 and GRACE (IH6mDthMI) > 161 were even better than those of HEART ≥8 and TIMI ≥5 without compromising specificity. As shown in Supplementary Table 4, GRACE (IHDthMI) > 168 and GRACE (IH6mDthMI) > 146 recognized 19% and 18% of patients as high risk, which were smaller than the proportion (22%) identified by the HEART score (7–10). However, the GRACE (IHDthMI) > 168 had greater specificity (0.904, 0.892–0.916) and PPV (0.591, 0.550–0.633) than HEART ≥7, with a specificity of 0.870 (0.856, 0.884) and a PPV of 0.534 (0.496, 0.573).

### Performance of the mini-GRACE models

The mini-GRACE (IHDthMI), mini-GRACE (IH6mDthMI), mini-GRACE (IHDth), mini-GRACE (IH6mDth) and mini-GRACE (OH6mDth) showed positive linear correlations with the actual MACE rates (r ≥ 0.793,* P* < 0.001). A very strong relationship remained in the mini-GRACE (IH6mDthMI) (r = 0.917). The mini-GRACE (IHDth), mini-GRACE (IH6mDth) and mini-GRACE (OH6mDth) had good calibration (*P* ≥ 0.517) while the other two did not (Supplementary Table 5). The mini-GRACE (IHDthMI) and mini-GRACE (IH6mDthMI) models, with AUCs of 0.82 (0.80, 0.83) and 0.81 (0.79, 0.82), respectively, were still superior to other models in discrimination and reclassification (Supplementary Figure 1) (Supplementary Table 6).

## Discussion

This study provides the first comprehensive evaluation and comparison of all six GRACE risk-prediction models in patients with undifferentiated chest pain. In the two Chinese EDs included in this study, all six GRACEs showed a positive linear correlation with actual MACE rates, and the five models had good calibration. All the C-statistics were ≥ 0.70. The GRACE (IHDthMI) and GRACE (IH6mDthMI) exhibited very strong relationships with actual MACE rates (r > 0.9) and showed excellent discriminatory capability (AUC > 0.80). Improvements in AUC, NRI and IDI indicated that GRACE (IHDthMI) and GRACE (IH6mDthMI) were comparable to the HEART score and superior to the other models.

The GRACE risk scores were developed using multivariable regression to assist cardiologists in estimating the risk of different outcomes in hospitalized patients with ACS and have been indicated to provide the most accurate stratification of risk of ACS both on admission and at discharge [31, 32]. One model is specific to one kind of outcome, including death or composite of MI and death during hospitalization, within 6 months after discharge and from admission to 6 months later. The MI referred to here is the subsequent AMI occurring after the index ACS. However, for undifferentiated chest pain, the high-risk conditions mainly present a composite endpoint of index AMI, subsequent AMI, death, emergency revascularization, cardiac arrest and cardiogenic shock within 30 days after presentation to the ED [33]. The incidence of index AMI is much greater than that of subsequent AMI, as shown in our study. Our results suggested that the GRACE models showed at least a moderate correlation with the actual incidence of MACEs in the undifferentiated chest pain cohort. In particular, very strong correlations appeared in GRACE (IHDthMI) and GRACE (IH6mDthMI). Furthermore, the predicted probabilities of an event and the observed event rates were significantly similar across deciles of five GRACE models. Therefore, there are foundations for the GRACE models to provide accurate stratification of patients with acute chest pain.

In previous studies, C-statistics for predicting 30-day MACEs in chest pain patients were merely evaluated according to the GRACE (IHDth) with AUCs of 0.60 to 0.83, which were always inferior to those of the HEART score [10–16]. Consistently, we found that the AUC of the GRACE (IHDth) was 0.75 (0.73, 0.76), which was actually lower than that of the HEART score in this study. However, GRACE (IHDth) was neither the only GRACE model nor the best GRACE model for stratifying chest pain. The GRACE (IHDthMI) and GRACE (IH6mDthMI) had better total discriminatory capability (AUC > 0.8) and reclassification without difference from the HEART score. Although the performance of all these models was not good in the rural hospital as in the urban hospital, the advantages of GRACE (IHDthMI), GRACE (IH6mDthMI) and HEART were consistent in both EDs. Significantly positive NRI and IDI in this study showed that the GRACE (IHDthMI) and GRACE (IH6mDthMI) could provide a higher predicted probability of an event for high-risk patients and a lower predicted probability for low-risk patients than the other four models. The possible explanation may be that events predicted by these two GRACE models referred to attacks of AMI rather than merely death, though not the index AMI. Compared with the models for events after discharge, the periods in the hospital or from admission to 6 months later were closer to the 30-day follow-up after presentation to the ED. Our results did not refute previous conclusions but complemented them by providing more complete recognition of the GRACE models.

Exact cutoff values should be determined for clinical use to identify low-risk patients for safe and early discharge without compromising the immediate treatment of high-risk chest pain. Reaney et al. found that GRACE (IHDth) 0–55 could reach a sensitivity of 95.2% and NPV of 95.8%, identifying 21.2% patients as low risk. GRACE (IHDth) ≥119 defined 16% of patients as high risk (specificity 89.8%; PPV 48.1%) [16]. Poldervaart et al. determined GRACE (IHDth) ≤72 as the cutoff and 19.1% patients were classified as low-risk (sensitivity 95%; NPV 96%) [14]. Cullen et al. chose the cutoff of GRACE (OH6mDth) ≤50 to determine low-risk (24% patients) with a sensitivity of 98.9%, and the cutoff for recognizing high risk (28% patients) was ≥100, with a specificity of 76.2% [17]. In our study, the performance of GRACE (IHDth) was relatively consistent with that of previous studies, with a value of ≤79 identifying 18% patients as low risk (sensitivity 95.1%; NPV 94.4%) and a value of > 145 defining 16% patients as high risk (specificity 90.0%; PPV 49.9%). At the same sensitivity and specificity, the GRACE (IHDthMI) and GRACE (IH6mDthMI) outperformed the GRACE (IHDth) and other GRACEs. Although there is no rigorous standard for the sensitivity of risk-stratification models for chest pain, an international survey suggested that clinicians may expect a sensitivity of 99% or higher for AMI or other MACEs [34]. If the sensitivity was set at ≥99%, GRACE (IHDthMI) and GRACE (IH6mDthMI) were still superior, but the proportions of low-risk patients would drop below 10%. A meta-analysis demonstrated that the pooled sensitivity and specificity of a HEART score ≤ 3 for predicting MACEs were 96.7% (94.0, 98.2%) and 47.0% (41.0, 53.5%), respectively [35]. HEART≤3 in our cohort had a similar sensitivity (96.8%) but a lower specificity (27.0%). The sensitivity of HEART ≤2 was higher at 98.8%(97.9, 99.7%) at the cost of a lower proportion (11%) of patients identified as low risk. In our previous report, the HEART score would not appear to provide additionally helpful risk stratification to the usual care for discharging low-risk patients [36]. Regarding the high-risk category, HEART ≥7 did not perform as well (specificity 87.0%; PPV 53.4%) as in Reaney’s study [16].

For ruling out and ruling in MACEs, the HEART score illustrated a certain advantage over the GRACE (IHDth) but not the GRACE (IHDthMI) or GRACE (IH6mDthMI). The strengths of GRACE are still noteworthy. Possible explanations might be the detailed class and objectivity of components of the GRACE beyond HEART and TIMI. Although the HEART score was directly developed for undifferentiated patients, the assignment of every variable only included three qualitative classes (i.e., 0,1,2) [21]. The classes for each component of TIMI score are even lesser (only 0 or 1) [22]. In contrast, the GRACE scores included many more quantitative variables, such as age, SBP, pulse and creatinine, which are supposed to identify subtler differences and result in more exact stratification. As highlighted by the 0 h/1 h algorithm from the ESC guideline recommendations, quantitative interpretation overcomes the qualitative interpretation of high-sensitivity troponin levels for ruling out and ruling in AMI in chest pain patients, and the cutoff levels are assay specific [6, 37]. Furthermore, some “soft” variables are included in the HEART score, such as the medical history, risk factors and symptoms. It has been shown that these variables do not have a sufficient discriminatory ability to rule in or rule out ACS in the ED [38]. The combination of symptom variables as a “history” component in the HEART score was still not clearly stated and not assessed systematically [39]. The GRACE score can avoid this situation due to the absence of subjective variables. The popularity of handheld devices has made the complexity of GRACE no longer a disadvantage.

The results from the assessment of mini-GRACE were mainly in accordance with those of the complete models. Although the correlation of the mini-GRACE (IHDthMI) and the calibration of the mini-GRACE (IH6mDthMI) were lower than the complete models, the discrimination and reclassification of these two mini scores remained excellent and significantly outperformed other models. This illustrates that the differences in model performance may be due to the disparities in weights of shared variables.

### Limitations

This study had several limitations. First, the performance of different GRACE scores was assessed in chest pain patients from two hospitals in China. Although urban and rural hospitals were both included, the validation of each score in wider patients should be determined by further studies of heterogeneous groups. In particular, the cutoff levels of the GRACEs are not the same in different studies due to the disparity of inclusion and exclusion criteria and the incidence and definition of MACEs. Determination and validation of the specific cutoff values in clinical practice in certain hospitals are needed. Second, the cardiac marker used in the calculation of scores was the contemporary cTn assay arranged by emergency physicians in their daily work. The ability of scores combined with high-sensitivity cTns to stratify chest pain still needs to be evaluated in future studies. Third, all components used in the risk scores were calculated automatically through a computer algorithm. The ECG variables were based on the standard interpretation from senior cardiologists. This calculation process deviated from clinical reality. Further studies to evaluate the discrimination of scores calculated immediately by the treating physicians are needed.

## Conclusions

From our evaluation and comparison of the six GRACE models in a prospective cohort of undifferentiated chest pain patients in two Chinese EDs, we found that all six GRACE models presented acceptable or excellent discriminatory capacity for predicting 30-day MACEs. In particular, the GRACE (IHDthMI) and GRACE (IH6mDthMI) were comparable to the HEART score and superior to other GRACEs and TIMI in terms of discrimination and reclassification. At a certain sensitivity and specificity, GRACE (IHDthMI) and GRACE (IH6mDthMI) could identify more patients to rule out or rule in 30-day MACEs than other models. Although cutoff levels of the GRACEs may be specific in different cohorts and validation of these levels are needed, the reasonable application of appropriate GRACE models in the evaluation of undifferentiated chest pain patients in ED should be recommended.

## Supplementary information

**Additional file 1: Figure S1.** Receiver operating characteristic curves of mini-GRACE models to predict MACEs within 30 days. *GRACE* Global Registry of Acute Coronary Events, *MACEs* major adverse cardiac events. * Significantly different from the HEART score. # Significantly different from the TIMI score. **Table S1.** Components and assignments of the HEART and TIMI scores. **Table S2.** C-statistics of GRACE models, HEART and TIMI scores to predict MACEs within 30 days in each participating hospital. **Table S3.** Performance of different models in terms of diagnostic accuracy at cutoff values with certain sensitivities. **Table S4.** Performance of different models in terms of diagnostic accuracy at cutoff values with specificity ≥90%. **Table S5.** Measures of the correlation and calibration of the mini-GRACE models. **Table S6.** The reclassification measurements of the mini-GRACE (IHDthMI) and mini-GRACE (IH6mDthMI) compared with other scores.

## Data Availability

The datasets used and analyzed during the current study are available from the corresponding author on reasonable request.

## Abbreviations

ACS: Acute coronary syndrome
AMI: Acute myocardial infarction
AUC: Area under the curve
CIs: Confidence intervals
CRF: Case report form
ED: Emergency department
GRACE: Global registry of acute coronary events
HEART: History, ECG, age, risk factors, troponin
HLT: Hosmer-Lemeshow goodness-of-fit test
IDI: Integrated discrimination improvement
MACEs: Major adverse cardiac events
NRI: Net reclassification improvement
NPV: Negative predictive value
PPV: Positive predictive value
STEMI: ST-elevation myocardial infarction
TIMI: Thrombolysis in myocardial infarction
URL: Upper reference limit
