# Supplementary material for: Evaluation and comparison of six GRACE models for the stratification of undifferentiated chest pain in the emergency department
Source: BMC Cardiovasc Disord. 2020 Apr 25;20:199. doi: 10.1186/s12872-020-01476-3 (PMC7183650; doi:10.1186/s12872-020-01476-3)
Supplement: Supplementary file 1 — Additional file 1: Figure S1. Receiver operating characteristic curves of mini-GRACE models to predict MACEs within 30 days. GRACE Global Registry of Acute Coronary Events, MACEs major adverse cardiac events. * Significantly different from the HEART score. # Significantly different from the TIMI score. Table S1. Components and assignments of the HEART and TIMI scores. Table S2. C-statistics of GRACE models, HEART and TIMI scores to predict MACEs within 30 days in each participating hospital. Table S3. Performance of different models in terms of diagnostic accuracy at cutoff values with certain sensitivities. Table S4. Performance of different models in terms of diagnostic accuracy at cutoff values with specificity ≥90%. Table S5. Measures of the correlation and calibration of the mini-GRACE models. Table S6. The reclassification measurements of the mini-GRACE (IHDthMI) and mini-GRACE (IH6mDthMI) compared with other scores. [file 12872_2020_1476_MOESM1_ESM.docx]

**Supplementary Table 1 Components and assignments of the HEART and TIMI scores**

| **HEART** | | |
| --- | --- | --- |
| **History** | Highly suspicious | 2 |
|  | Moderately suspicious | 1 |
|  | Slightly suspicious | 0 |
| **ECG** | New ischaemic changes | 2 |
|  | Non-specific changes | 1 |
|  | Normal | 0 |
| **Age** | ≥65 | 2 |
|  | 45-64 | 1 |
|  | <45 | 0 |
| **Risk factors** | ≥3 cardiac risk factors or history of atherosclerotic disease | 2 |
|  | 1 or 2 risk factors | 1 |
|  | No risk factors known | 0 |
| **Troponin** | >3×ULN | 2 |
|  | 1-3×ULN | 1 |
|  | ≤ULN | 0 |
| **TIMI** | | |
| **Age** | ≥65 | 1 |
| **Risk factors** | ≥3 cardiac risk factors | 1 |
| **Medical history** | Prior coronary stenosis | 1 |
| **ECG** | ST-segment deviation | 1 |
| **Symptom** | Severe angina symptom | 1 |
| **Medication** | Use of aspirin in last 7days | 1 |
| **Troponin** | Elevated cardiac troponin | 1 |

*ECG* electrocardiography, *HEART* History, ECG, Age, Risk factors, Troponin, *TIMI* Thrombolysis in Myocardial Infarction, *ULN* upper limit of normal

**Supplementary Table 2** C-statistics of GRACE models, HEART and TIMI scores to predict MACEs within 30 days in each participating hospital

|  | **AUC (95%CI)** | |
| --- | --- | --- |
|  | **Urban** | **Rural** |
| GRACE (IHDthMI) | 0.844 (0.829, 0.858) # | 0.723 (0.680, 0.763) # |
| GRACE (IH6mDthMI) | 0.837 (0.822, 0.851) # | 0.720 (0.677, 0.760) # |
| GRACE (IHDth) | 0.755 (0.738, 0.772)* | 0.683 (0.639, 0.725)* |
| GRACE (IH6mDth) | 0.738 (0.720, 0.756)* | 0.686 (0.642, 0.728)* |
| GRACE (OH6mDth) | 0.726 (0.708, 0.744)* | 0.675 (0.631, 0.717)* |
| GRACE (OH6mDthMI) | 0.716 (0.697, 0.734)* | 0.668 (0.624, 0.711)* |
| HEART | 0.836 (0.820, 0.850) # | 0.737 (0.695, 0.777) # |
| TIMI | 0.736 (0.718, 0.753)* | 0.649 (0.604, 0.692)* |

*CI* confidence interval, *GRACE* Global Registry of Acute Coronary Events, *HEART* History, ECG, Age, Risk factors, Troponin, *MACE* major adverse cardiac event, *TIMI* Thrombolysis in Myocardial Infarction

* Significantly different from the HEART score

# Significantly different from the TIMI score

**Supplementary Table 3** Performance of different models in terms of diagnostic accuracy at cutoff values with certain sensitivities

| **Scores and cutoff values** | **Low-risk** | **Sensitivity**  **(95%CI)** | **NPV**  **(95%CI)** | **Specificity**  **(95%CI)** | **PPV**  **(95%CI)** |
| --- | --- | --- | --- | --- | --- |
| **Sensitivity ≥98%** |  |  |  |  |  |
| GRACE (IHDthMI) ≤64 | 407 (14%)*# | 0.980(0.968,0.991) | 0.971(0.954,0.987) | 0.172(0.157,0.187)* # | 0.233(0.217,0.250) |
| GRACE (IH6mDthMI) ≤61 | 411 (14%)*# | 0.980(0.968,0.991) | 0.971(0.955,0.987) | 0.174(0.158,0.189)* # | 0.234(0.217,0.250) |
| GRACE (IHDth) ≤68 | 280 (10%)# | 0.981(0.970,0.992) | 0.961(0.938,0.983) | 0.117(0.104,0.130) # | 0.222(0.206,0.238) |
| GRACE (IH6mDth) ≤50 | 290 (10%)# | 0.981(0.970,0.992) | 0.962(0.940,0.984) | 0.122(0.108,0.135) # | 0.223(0.207,0.239) |
| GRACE (OH6mDth) ≤60 | 281 (10%)# | 0.981(0.970,0.992) | 0.961(0.938,0.984) | 0.118(0.104,0.131) # | 0.222(0.206,0.238) |
| GRACE (OH6mDthMI) ≤59 | 275 (10%)# | 0.981(0.970,0.992) | 0.960(0.937,0.983) | 0.115(0.102,0.128) # | 0.222(0.206,0.238) |
| HEART ≤2 | 305 (11%) | 0.988(0.979,0.997) | 0.977(0.960,0.994) | 0.130(0.116,0.144) # | 0.226(0.210,0.242) |
| TIMI =0 | 351 (12%) | 0.986(0.977,0.996) | 0.977(0.962,0.993) | 0.149(0.135,0.164)* | 0.230(0.213,0.246) |
| **Sensitivity ≥99%** |  |  |  |  |  |
| GRACE (IHDthMI) ≤53 | 198 (7%)* | 0.993(0.987,1.000) | 0.980(0.960,0.999) | 0.084(0.073,0.096)* | 0.218(0.202,0.234) |
| GRACE (IH6mDthMI) ≤51 | 255 (9%)* | 0.992(0.984,0.999) | 0.980(0.963,0.997) | 0.109(0.096,0.122)* | 0.222(0.206,0.238) |
| GRACE (IHDth) ≤62 | 203 (7%)* | 0.992(0.984,0.999) | 0.975(0.954,0.997) | 0.086(0.075,0.098)* | 0.218(0.202,0.234) |
| GRACE (IH6mDth) ≤43 | 197 (7%)* | 0.992(0.984,0.999) | 0.975(0.953,0.997) | 0.084(0.072,0.095)* | 0.218(0.202,0.233) |
| GRACE (OH6mDth) ≤57 | 241 (8%)* | 0.992(0.984,0.999) | 0.979(0.961,0.997) | 0.103(0.090,0.115)* | 0.221(0.205,0.237) |
| GRACE (OH6mDthMI) ≤55 | 220 (8%)* | 0.993(0.987,1.000) | 0.982(0.964,0.999) | 0.094(0.082,0.106)* | 0.220(0.204,0.236) |
| HEART ≤1 | 93 (3%) | 1.000(0.994,1.000) | 1.000(1.000,1.000) | 0.041(0.032,0.049) | 0.211(0.196,0.226) |

*CI* confidence interval, *GRACE* Global Registry of Acute Coronary Events, *HEART* History, ECG, Age, Risk factors, Troponin, *MACE* major adverse cardiac event, *NPV* negative predictive value, *PPV* positive predictive value, *TIMI* Thrombolysis in Myocardial Infarction

* Significantly different from the HEART score

# Significantly different from the TIMI score

**Supplementary Table 4** Performance of different models in terms of diagnostic accuracy at cutoff values with specificity ≥90%

| **Scores and cutoff values** | **High-risk** | | **Sensitivity**  **(95%CI)** | | **NPV**  **(95%CI)** | | **Specificity**  **(95%CI)** | **PPV**  **(95%CI)** |
| --- | --- | --- | --- | --- | --- | --- | --- | --- |
| **Specificity ≥90%** | |  | |  |  |  | |  |
| GRACE (IHDthMI) >168 | | 541 (19%)* | | 0.542(0.502,0.583) | 0.885(0.872,0.898) | 0.904(0.892,0.916)* | | 0.591(0.550,0.633)* |
| GRACE (IH6mDthMI) >146 | | 531 (18%)* | | 0.517(0.477,0.557)* | 0.879(0.866,0.892) | 0.902(0.889,0.914)* | | 0.574(0.532,0.616) |
| GRACE (IHDth) >145 | | 457 (16%)* | | 0.386(0.347,0.426)* | 0.851(0.837,0.865)* | 0.900(0.888,0.913)* | | 0.499(0.453,0.545) |
| GRACE (IH6mDth) >121 | | 428 (15%)* | | 0.361(0.322,0.400)* | 0.847(0.832,0.861)* | 0.906(0.894,0.918)* | | 0.498(0.450,0.545) |
| GRACE (OH6mDth) >132 | | 413 (14%)* | | 0.332(0.294,0.370)* | 0.841(0.826,0.855)* | 0.905(0.894,0.917)* | | 0.475(0.426,0.523) |
| GRACE (OH6mDthMI) >135 | | 382 (13%)* | | 0.275(0.239,0.311)* | 0.829(0.814,0.844)* | 0.904(0.892,0.916)* | | 0.424(0.375,0.474)* |
| HEART ≥7 | | 640 (22%) | | 0.580(0.540,0.619) | 0.890(0.877,0.903) | 0.870(0.856,0.884) | | 0.534(0.496,0.573) |

*CI* confidence interval, *GRACE* Global Registry of Acute Coronary Events, *HEART* History, ECG, Age, Risk factors, Troponin, *MACE* major adverse cardiac event, *NPV*, negative predictive value, *PPV*, positive predictive value

* Significantly different from the HEART score

**Supplementary Table 5** Measures of the correlation and calibration of the mini-GRACE models

|  | **Correlation** | | **Calibration** | |
| --- | --- | --- | --- | --- |
|  | **r** | ***P* value** | **HLT χ2** | ***P* value** |
| mini-GRACE (IHDth) | 0.857 | <0.001 | 1.5991 | 0.991 |
| mini-GRACE (IHDthMI) | 0.891 | <0.001 | 47.8725 | <0.001 |
| mini-GRACE (OH6mDth) | 0.793 | <0.001 | 6.6532 | 0.575 |
| mini-GRACE (IH6mDth) | 0.876 | <0.001 | 7.1873 | 0.517 |
| mini-GRACE (IH6mDthMI) | 0.917 | <0.001 | 15.937 | 0.043 |

*GRACE* Global Registry of Acute Coronary Events, *HLT* Hosmer-Lemeshow goodness-of-fit test

**Supplementary Table 6** The reclassification measurements of the mini-GRACE (IHDthMI) and mini-GRACE (IH6mDthMI) compared with other scores

|  | **NRI (95%CI)** | ***P* value** | **IDI (95%CI)** | ***P* value** |
| --- | --- | --- | --- | --- |
| **mini-GRACE (IHDthMI) vs** | | | | |
| mini-GRACE (IHDth) | 0.938 (0.863, 1.014) | <0.001 | 0.136 (0.125, 0.148) | <0.001 |
| mini-GRACE (IH6mDth) | 0.831 (0.751, 0.911) | <0.001 | 0.153 (0.140, 0.166) | <0.001 |
| mini-GRACE (OH6mDth) | 0.795 (0.713, 0.876) | <0.001 | 0.159 (0.144, 0.174) | <0.001 |
| GRACE (OH6mDthMI) | 0.733 (0.650, 0.817) | <0.001 | 0.179 (0.161, 0.197) | <0.001 |
| **mini-GRACE (IH6mDthMI) vs** | | | | |
| mini-GRACE (IHDth) | 1.097 (1.019, 1.176) | <0.001 | 0.111 (0.102, 0.121) | <0.001 |
| mini-GRACE (IH6mDth) | 1.050 (0.974, 1.126) | <0.001 | 0.127 (0.117, 0.138) | <0.001 |
| mini-GRACE (OH6mDth) | 0.911 (0.830, 0.991) | <0.001 | 0.134 (0.121, 0.146) | <0.001 |
| GRACE (OH6mDthMI) | 0.824 (0.741, 0.906) | <0.001 | 0.154 (0.138, 0.170) | <0.001 |

*CI* confidence interval, *GRACE* Global Registry of Acute Coronary Events, *IDI* integrated discrimination improvement, *NRI* net reclassification improvement

**
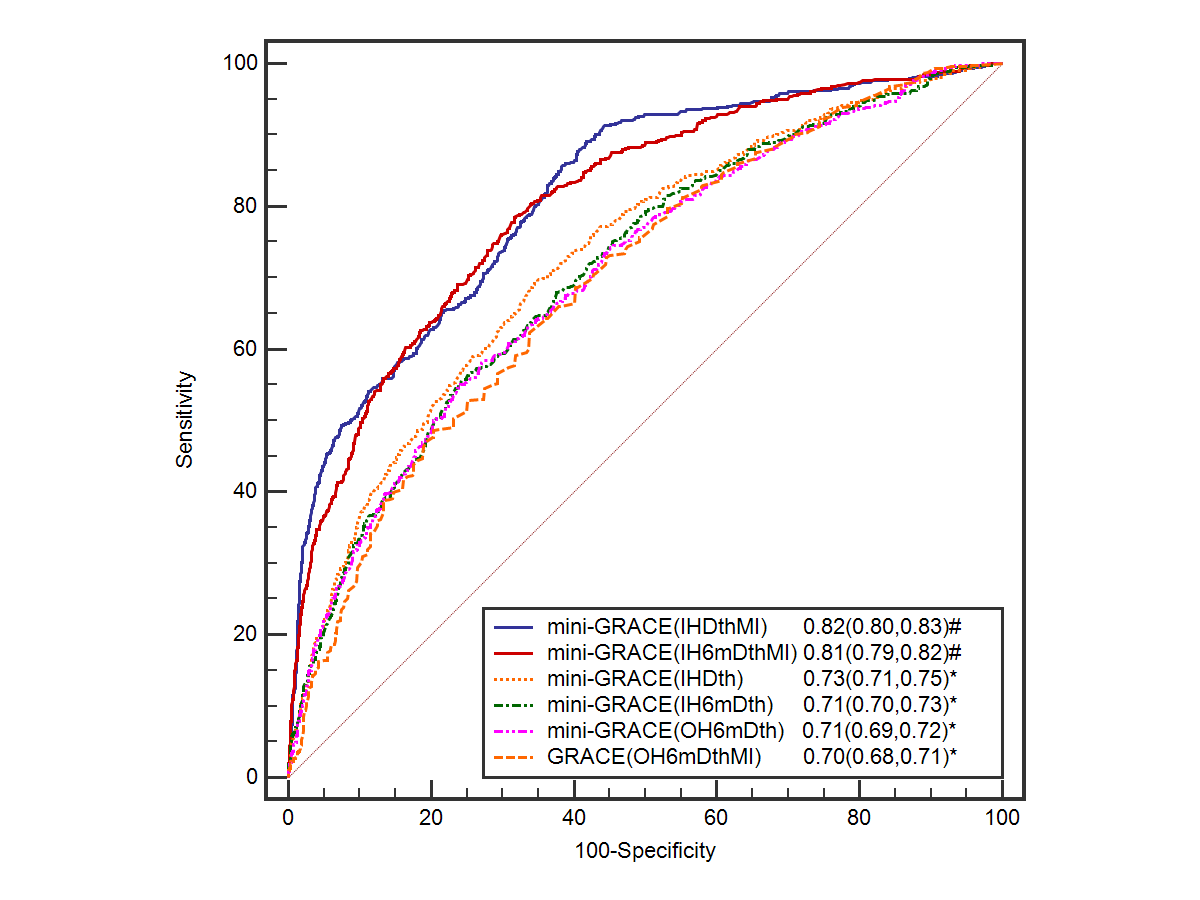
**

**Supplementary Figure 1** Receiver operating characteristic curves of mini-GRACE models to predict MACEs within 30 days

*GRACE* Global Registry of Acute Coronary Events, *MACEs* major adverse cardiac events

* Significantly different from the HEART score

# Significantly different from the TIMI score
